# Bigger, Brighter, Bluer-Better? Current Light-Emitting Devices – Adverse Sleep Properties and Preventative Strategies

**DOI:** 10.3389/fpubh.2015.00233

**Published:** 2015-10-13

**Authors:** Paul Gringras, Benita Middleton, Debra J. Skene, Victoria L. Revell

**Affiliations:** ^1^Department of Children’s Sleep Medicine, Evelina London Children’s Sleep Medicine and King’s College London, St Thomas’ Hospital, London, UK; ^2^Faculty of Health and Medical Sciences, University of Surrey, Guildford, UK

**Keywords:** sleep disorders, light, tablets, smartphone, apps, melatonin

## Abstract

**Objective:**

In an effort to enhance the efficiency, brightness, and contrast of light-emitting (LE) devices during the day, displays often generate substantial short-wavelength (blue-enriched) light emissions that can adversely affect sleep. We set out to verify the extent of such short-wavelength emissions, produced by a tablet (iPad Air), e-reader (Kindle Paperwhite 1st generation), and smartphone (iPhone 5s) and to determine the impact of strategies designed to reduce these light emissions.

**Setting:**

University of Surrey dedicated chronobiology facility.

**Methods:**

First, the spectral power of all the LE devices was assessed when displaying identical text. Second, we compared the text output with that of “Angry Birds” – a popular top 100 “App Store” game. Finally, we measured the impact of two strategies that attempt to reduce the output of short-wavelength light emissions. The first strategy employed an inexpensive commercially available pair of orange-tinted “blue-blocking” glasses. The second strategy tested an app designed to be “sleep-aware” whose designers deliberately attempted to reduce short-wavelength light emissions.

**Results:**

All the LE devices shared very similar enhanced short-wavelength peaks when displaying text. This included the output from the backlit Kindle Paperwhite device. The spectra when comparing text to the Angry Birds game were also very similar, although the text emissions were higher intensity. Both the orange-tinted glasses and the “sleep-aware” app significantly reduced short-wavelength emissions.

**Conclusion:**

The LE devices tested were all bright and characterized by short-wavelength enriched emissions. Since this type of light is likely to cause the most disruption to sleep as it most effectively suppresses melatonin and increases alertness, there needs to be the recognition that at night-time “brighter and bluer” is not synonymous with “better.” Ideally future software design could be better optimized when night-time use is anticipated, and hardware should allow an automatic “bedtime mode” that shifts blue and green light emissions to yellow and red as well as reduce backlight/light intensity.

## Background and Objectives

A growing body of evidence suggests that the use of light-emitting (LE) devices in the evening may adversely affect sleep quality and timing, daytime performance, health, and safety (1–3). The brightness, timing, color, pattern, and the duration of light exposure all influence important physiological body rhythms (4–6). When modern LE devices are used in the evening before bedtime all these factors combine to produce a “perfect storm,” which can adversely affect sleep.

The role of light and its influence on many aspects of our physiology, behavior and well-being is increasingly well understood (4–6). In particular, the light/dark cycle is critical in synchronizing the circadian (daily) clock to the 24 h day. The hormone melatonin (“the hormone of darkness”) is produced at night, with the duration of secretion mirroring the dark period, and its production is associated with sleep (7).

While light during the daytime can beneficially enhance alertness, performance, and mood (8), in the evening it can suppress the production of melatonin, increase alertness, and delay sleep onset (9).

Importantly, not all colors of light have the same effect. Short-wavelength-enriched light (blue-enriched) is likely to cause the most disruption, as it most effectively suppresses melatonin (10) and increases alertness (11). Many older LE devices have been shown to have peaks specifically in these short wavelengths (3).

The development of LE devices means that for many people, a “book at bedtime” is now often an “e-book.” Traditional paper books with dim incandescent bedside lighting reflected off the pages of the book expose the readers to a low-intensity tungsten light with a yellow–red spectrum that has little impact on sleep. In comparison, the same book read in electronic format will provide a very different light signal with biological effects. This is not an insignificant issue with over a quarter of the US population reading e-books in 2014 (12). Furthermore, these same LE devices allow access to the Internet, social media, and games as well as reading, with evidence that multi-tasking is becoming the norm rather than the exception (13).

Studies considering the potential impact of light exposure at night have employed a variety of methodologies, including animal studies (14), laboratory-based controlled-environment studies (3–6), and epidemiological studies (13, 15). All have important roles, with advantages and limitations.

Until 2000, the majority of photometric studies quantified light stimuli in terms of photopic illuminance (lux) (16). During that time, inexpensive lux meters were used because of their existing role in lighting and photography. As the existence and role of melanopsin and the intrinsically photosensitive retinal ganglion cells (ipRGC) in the inner retina have become clearer, so has the realization that current methods of light measurement are incomplete (16). In order to better characterize the biological effects of light, a “toolkit” to calculate the effective irradiance experienced by each of the rod, cone, and melanopsin photoreceptors has been developed (16).

We set out to measure light levels and spectral profiles of three of the most popular contemporary LE devices to verify and compare their short-wavelength-enriched light emissions. We decided to include three categories of devices; one tablet, one smartphone, and one e-reader. As we were not aware of studies comparing activities such as reading an e-book with playing a game, we also compared the light signals emitted when playing a popular game, with those emitted by e-book text.

Since there are a number of potential strategies that claim to reduce the intensity of short-wavelength light exposure, we also sought to test the actual effect of some of these strategies on the spectral profile of these light emissions.

By characterizing the extent to which each of the five photopigments in the human eye are activated by all the light conditions we tested, we intended to provide reliable benchmark data for each LE device, to allow later comparison with other devices, other conditions, and extrapolation to physiological and behavioral responses.

## Materials and Methods

Using data from International Data Corporation (IDC) (17) and Canaccord Genuity (18), we chose the most popular devices of 2014 (iPad Air, iPhone 5s, Kindle Paperwhite 1st generation) from each of the three categories (tablets, smartphones, e-readers).

Since all the devices claim to be easily viewed at night in a dark room without any room lighting, we carried out spectrometric light readings in the same completely dark room. Light measurements were taken at a distance suggested as a typical reading distance, as advised by each device’s manufacturer (Table 1).

**Table 1:** **Physical properties of LE devices tested**.

	Kindle Paperwhite (1st generation B020)	iPhone 5S (A1453)	iPad Air (A1474)
Screen diagonal (inches)	6	4	9.7
Pixel per inch	212	326	264
Technology	E Ink Carta/LED frontlit	LED-backlit with IPS Technology	LED-Backlit with IGZO technology
Distance measured (cm)	35	22.5	35

*The iPad air and iPhone 5S are registered trademarks of Apple Inc. The Kindle and Kindle store are registered trademarks of Amazon.com. Angry Birds is a registered trademark of Rovio Mobile*.

The brightness levels of the screens were not adjusted for those devices that had an automatic setting (iPhone 5s and ipad Air) but the Kindle Paperwhite screen brightness level was reduced to 50% (guided by “typical night time settings” feedback from a convenience sample of 10 Kindle Paperwhite users). For each device, the irradiance as an exact spectral power distribution (SPD) was measured using the same calibrated spectrometer (Ocean Optics BV, Dunedin, FL, USA).

During e-book measurements, identical text was displayed across all devices – the same page, of the same downloaded e-book via the Kindle store. (We had previously looked at increasing and decreasing the font size within the boundaries available on the Kindle reading app and this made no significant difference to the light irradiance measurements.) For the game measurements, we used the same screen from “Angry Birds” – a popular top 100 “App Store” game by Rovio mobile (this was displayed on the iPad Air and iPhone 5s but not on the Kindle Paperwhite as this device does not display games).

These measurements were followed by testing the two different strategies designed to reduce the output of short-wavelength enriched light emissions. The first strategy involved testing the impact of blue-blocking (orange-tinted) glasses (Pyramex Ztek Safety Eyewear). These were tested by holding one of the lenses of the glasses 2.5 cm in front of the spectrometer probe. The second tested “Kids Sleep Dr” (a sleep diary/behavioral advice app intended to help parents solve their children’s sleep problems) (19). As this app is designed to be used during the evening and at night, its developers took this into account and chose a “sleep-aware” palate of colors accordingly. This is the only app we were aware of at the time that employed this strategy.

Finally, using the measured SPD, we calculated the equivalent “α-opic” illuminance for each of the five photopigments in the human eye using the recently proposed light measurement strategy (16).

## Results

Table 2 displays the device emission spectra (cyanopic, melanopic, rhodopic, chloropic and erythopic “α-opic” lux) in comparison with photopic lux for all LE devices and conditions.

**Table 2:** **Spectral distribution of human retinal photopigment-weighted measures from all light-emitting devices during different display conditions**.

Prefix	Sensitivity	***α***-opic lux						
		Angry Birds ipad	Angry birds phone	Kids sleep Dr	Text ipad	Text ipad glasses	Text kindle	Text phone
Cyanopic	S cone	244.44	63.03	27.68	409.18	59.23	46.95	71.52
Melanopic	Melanopsin	176.25	46.49	31.51	302.33	64.55	34.62	54.54
Rhodopic	Rod	180.07	45.04	39.65	313.43	93.68	35.64	53.92
Chloropic	M cone	174.03	41.96	71.55	314.00	154.16	37.56	52.04
Erythropic	L cone	162.66	39.72	112.96	306.52	199.93	37.68	50.49
Photopic lux	lux	170.42	40.32	104.95	318.52	201.89	38.67	51.40
Irradiance	μ W/cm^2^	60.20	16.40	39.10	110.80	62.30	14.30	19.80
Photon flux	1/cm^2^/s	1.61E+14	4.41E+13	1.18E+14	3.00E+14	1.85E+14	3.90E+13	5.35E+13
Peak spectral irradiance	nm	445	450	610	445	605	455	450

*The ability of the light devices to stimulate the human photopigments in the eye was assessed and is presented in this table*.

*The potential ability of each light source to stimulate the S-cone (cyanopic), M-cone (chloropic), L-cone (erythopic), rods (rhodopic), and melanopsin (melanopic) photopigments, corrected for pre-receptoral filtering, was evaluated (16). The S-, M-, and L-cones make up the trichromatic visual system and melanopsin is the blue light sensitive irradiance detecting photopigment that is the primary contributor to the non-visual responses to light*.

*Light intensity irradiance is measured in microwatt per square centimeter. Photon flux is the number of photons that get delivered by the device per square centimeter per second*.

*Peak spectral irradiance is the wavelength (nanometer) of the peak where the irradiance is highest*.

**Figure 1:**
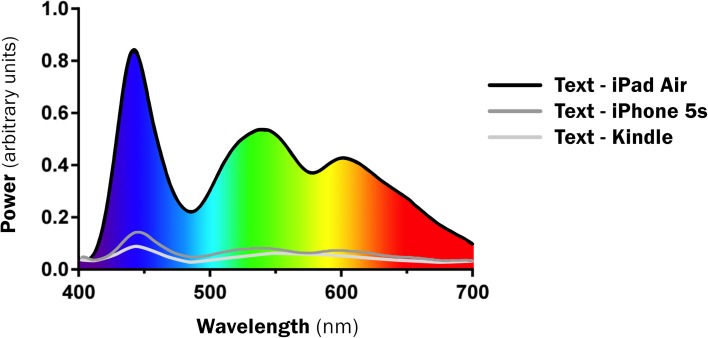
**Spectral Profile comparing identical text on all three devices**.

**Figure 2:**
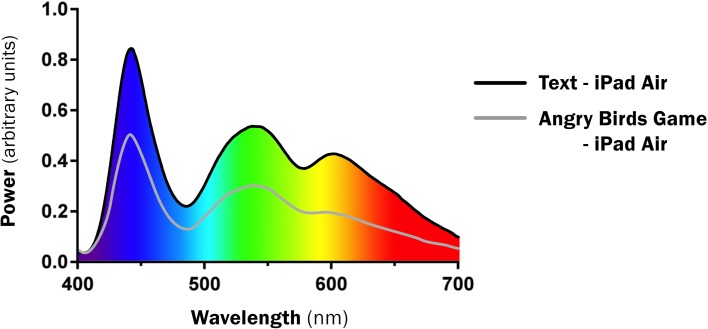
**Spectral Profile of Text compared to game (same device)**.

Interestingly, the LE devices all shared very similar enhanced short-wavelength blue peaks when displaying the same text (445–455 nm). This includes the output from the new backlit Kindle device. Figure 1 presents the SPD of text of all devices. The spectral profile of text and Angry Birds game were also very similar, although the text emissions were higher intensity (Figure 2).

**Figure 3:**
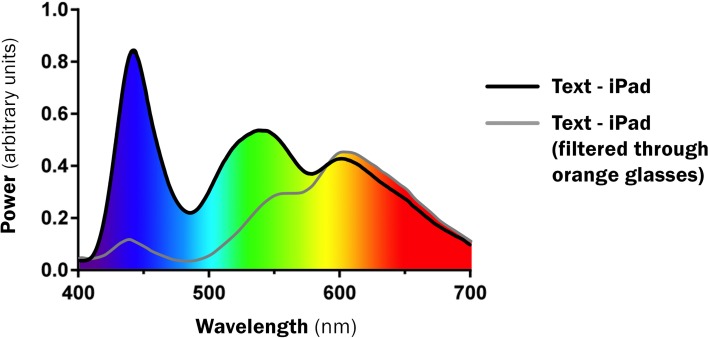
**Spectral Profile demonstrating impact of ‘blue-blocking’ glasses**.

**Figure 4:**
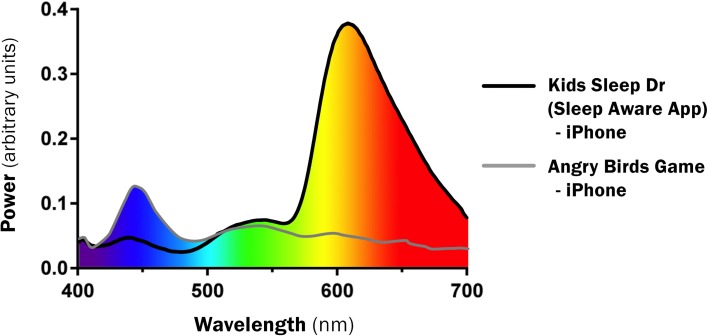
**Spectral Profile comparing ‘sleep aware’ designed game with normal game**.

The orange-tinted glasses significantly reduced short-wavelength light emissions (Figure 3). The color palate used in the Kids Sleep Dr app generated a different spectral profile to the “text” or “Angry Birds” with a marked reduction in short-wavelength light emissions (Figure 4).

## Discussion

We have shown that the “latest models” of tablet and smartphone LE devices that were tested maintained the same enhanced short-wavelength (blue-light) emitting characteristics as their predecessors (3). A third e-reader device (Kindle), previously non-backlit, is now backlit, and also emits the same short-wavelength (blue-enriched) light emissions.

Light levels emitted by games have not been previously compared with e-books, although the stimulating effects of games at night time have been documented (20). Interestingly, the game we tested had a very similar blue-enriched spectral profile to text, but was less bright.

There are some fairly simple strategies to reduce exposure to short-wavelength lighting before bed. The most obvious is to avoid exposure to light-emitting devices at night. Harvard Medical School suggest avoiding blue-light 2–3 h before you go to bed, while the National Sleep Foundation suggest turning all electronic devices off at least an hour before bed. Parents who have young children using LE Devices at night have the ability to either remove the devices from their bedroom, or at least turn them before bed. Falbe et al. (15) showed the associations between small screens in the sleep environment, screen time and shorter, insufficient sleep in school aged children. Such “removal” strategies become more difficult to implement with adolescents and adults, who make their own choices and are often influenced by peer and work pressures.

The two fairly simple light-blocking strategies we evaluated both effectively reduced the emission of short-wavelength blue-enriched light. The glasses we used were inexpensive, mass-produced plastic orange-tinted glasses often advertised “for shift workers.” The effect of these on the emitted spectrum, however, was significant and in keeping with more expensive blue-blockers that have recently been shown to attenuate evening suppression of melatonin while viewing an LED computer screen (21). The designers of the “sleep-aware” app adapted its colors using basic principles without needing expensive concurrent spectrometer measurements. This simple strategy was effective in reducing short-wavelength emissions.

Perhaps a more viable alternative to expecting people to wear such orange glasses, or app developers to limit their color palate, is to use software to apply a “mask,” or filter to the device itself. As we only tested one example of each of the currently most popular LE devices, this unfortunately meant we did not test android devices; had we done so we would have been able to also test software such as F.lux (developed by Michael and Lorna Herf that adjusts a computer display’s color temperature according to its location and time of day). This software, however, was not available for the iPad, iPhone, nor is it applicable for the Kindle Paperlight.

One important limitation of this study is that a multitude of environmental factors may interact and may contribute to sleep disruption. Light duration is one important factor whereby even a blue-light source of lower intensity, if viewed long enough, could still suppress nocturnal melatonin levels and increase alertness. Exactly how long, for each intensity and each device type, is not known, but it is an important area for future research. We only tested one “typical brightness” setting at one distance for each device. Some users of iPad and iPhone devices might manually adjust light intensity or invert colors, so our findings are only applicable to those users that rely on the default automatic lighting settings. Future research needs to construct full intensity responses curves for each device and to characterize the extent to which each of the five photoreceptor channels are activated using the Toolkit from Lucas et al. (16), the way we have done in the present manuscript. Following this, investigating individual variations in the response to such light sources will be necessary.

Another obvious limitation to research involving new technology is that the rate of releasing new LE devices, outstrips the time it takes to publish new research. The result is that studies that evaluate contemporary hardware are out of date by the time they are published (much as the hardware examined in this publication is also “last years model”).

Not withstanding these limitations, the trend is clear and technological “advances” to date for LE devices, seem to have focused on designs that enhance their brightness, blueness, visibility and contrast during the day. Unfortunately, these are the same characteristics that in the evening are likely to worsen timing and quality of sleep, and reduce morning alertness.

Despite increasing availability of information about the possible risks from evening LE device use, it is often hard to encourage people to make better health choices. The National Sleep Foundation’s Sleep in America Poll found nine out of ten Americans reported using a technological device in the hour before bed (13).

Even if this topic was determined important enough to warrant individual, community-based or national public health interventions, such approaches are expensive, difficult to implement, and often unsuccessful (22). A faster and more tenable solution would be for manufacturers to ensure that software design is optimized when night-time use is anticipated, and all hardware devices allow an automatic “bedtime mode” that shifts blue and green light emissions to yellow and red as well as reduces backlight/light intensity.

We hope that as technology improves, “brighter” will not always be synonymous with “better.”

## Ethics Approval

As no human subjects were involved, none of the CONSORT, PRISMA, MOOSE, STARD or STROBE frameworks with which we are familiar, were applicable for this research.

## Data Sharing Statement

Additional raw spectrometer data used for this article and from testing other apps are available on request from corresponding author.

## Conflict of Interest Statement

Debra J. Skene, Victoria L. Revell, and Benita Middleton declare no support from any organization for the submitted work, no financial relationships with any organizations that might have an interest in the submitted work in the previous 3 years, no other relationships or activities that could appear to have influenced the submitted work. Paul Gringras was involved in the development of the Kids Sleep Dr app – one piece of software whose light emissions were measured in this research. However, he has received no financial support for this development, and the app is a free resource with IP owned by Guys and St Thomas NHS Foundation Trust.

## References

[1] Wood B Rea MS Plitnick B Figueiro MG. Light level and duration of exposure determine the impact of self-luminous tablets on melatonin suppression. Appl Ergon (2013) 44(2):237–40. 10.1016/j.apergo.2012.07.008 22850476

[2] Cajochen C Frey S Anders D Späti J Bues M Pross A Evening exposure to a light-emitting diodes (LED)-backlit computer screen affects circadian physiology and cognitive performance. J Appl Physiol (2011) 110(5):1432–8. 10.1152/japplphysiol.00165.2011 21415172

[3] Chang AM Aeschbach D Duffy JF Czeisler CA. Evening use of light-emitting eReaders negatively affects sleep, circadian timing, and next-morning alertness. Proc Natl Acad Sci U S A (2014) 112:18490. 10.1073/pnas.1418490112 25535358 PMC4313820

[4] Chang AM Santhi N St Hilaire M Gronfier C Bradstreet DS Duffy JF Human responses to bright light of different durations. J Physiol (2012) 590:3103–12. 10.1113/jphysiol.2011.226555 22526883 PMC3406393

[5] Czeisler CA Kronauer RE Allan JS Duffy JF Jewett ME Brown EN Bright light induction of strong (type 0) resetting of the human circadian pacemaker. Science (1989) 244(4910):1328–33. 10.1126/science.2734611 2734611

[6] Revell VL Barrett DC Schlangen LJ Skene DJ. Predicting human nocturnal nonvisual responses to monochromatic and polychromatic light with a melanopsin photosensitivity function. Chronobiol Int (2010) 27(9–10):1762–77. 10.3109/07420528.2010.516048 20969522

[7] Skene DJ Arendt J. Human circadian rhythms: physiological and therapeutic relevance of light and melatonin. Ann Clin Biochem (2006) 43:344–53. 10.1258/000456306778520142 17022876

[8] Phipps-Nelson J Redman JR Dijk DJ Rajaratnam SMW. Daytime exposure to bright light, as compared to dim light, decreases sleepiness and improves psychomotor vigilance performance. Sleep (2003) 26(6):695–700. 14572122 10.1093/sleep/26.6.695

[9] Santhi N Thorne HC van der Veen DR Johnsen S Mills SL Hommes V The spectral composition of evening light and individual differences in the suppression of melatonin and delay of sleep in humans. J Pineal Res (2012) 53:47–59. 10.1111/j.1600-079X.2011.00970.x 22017511

[10] Thapan K Arendt J Skene DJ. An action spectrum for melatonin suppression: evidence for a novel non-rod, non-cone photoreceptor system in humans. J Physiol (2001) 535:226–61. 10.1111/j.1469-7793.2001.t01-1-00261.x 11507175 PMC2278766

[11] Cajochen C Münch M Kobialka S Kräuchi K Steiner R Oelhafen P High sensitivity of human melatonin, alertness, thermoregulation, and heart rate to short wavelength light. J Clin Endocrinol Metab (2005) 90(3):1311–6. 10.1210/jc.2004-0957 15585546

[12] Pew Research Center. E-Reading Rises as Device Ownership Jumps. (2014). Available from: http://pewinternet.org/Reports/2014/E-Reading-Update.aspx

[13] Gradisar M Wolfson AR Harvey AG Hale L Rosenberg R Czeisler CA. The sleep and technology use of Americans: findings from the National Sleep Foundation’s 2011 Sleep in America poll. J Clin Sleep Med (2013) 9(12):1291–9. 10.5664/jcsm.3272 24340291 PMC3836340

[14] Hatori M Le H Vollmers C Keding SR Tanaka N. Inducible ablation of melanopsin-expressing retinal ganglion cells reveals their central role in non-image forming visual responses. PLoS One (2008) 3(6):e2451. 10.1371/journal.pone.0002451 18545654 PMC2396502

[15] Falbe J Davison KK Franckle RL Ganter C Gortmaker SL Smith L Sleep duration, restfulness, and screens in the sleep environment. Pediatrics (2015) 135(2):e367–75. 10.1542/peds.2014-2306 25560435 PMC4306800

[16] Lucas RJ Peirson SN Berson DM Brown TM Cooper HM Czeisler CA Measuring and using light in the melanopsin age. Trends Neurosci (2014) 37(1):1–9. 10.1016/j.tins.2013.10.004 24287308 PMC4699304

[17] Available from: http://www.idc.com/tracker/showproductinfo.jsp?prod_id=81

[18] Available from: http://www.businessinsider.com/apple-and-samsung-have-106-of-the-smartphone-industrys-profits-2014-5?IR=T15

[19] Available from: www.kidssleepdr.com

[20] Higuchi S Motohashi Y Liu Y Maeda A. Effects of playing a computer game using a bright display on presleep physiological variables, sleep latency, slow wave sleep and REM sleep. J Sleep Res (2005) 14(3):267–73. 10.1111/j.1365-2869.2005.00463.x 16120101

[21] van der Lely S Frey S Garbazza C Wirz-Justice A Jenni OG Steiner R Blue blocker glasses as a countermeasure for alerting effects of evening light-emitting diode screen exposure in male teenagers. J Adolesc Health (2015) 56(1):113–9. 10.1016/j.jadohealth.2014.08.002 25287985

[22] Cutler DM Behavioral health interventions: what works and why? In: Anderson NB Bulatao RA Cohen B, editors. National Research Council: Critical Perspectives on Racial and Ethnic Differences in Health in Late Life. Washington, DC: The National Academies Press (2004). p. 643–76. 20669464

